# Supplementary material for: Exposure to Secondhand Smoke Extract Increases Cisplatin Resistance in Head and Neck Cancer Cells
Source: Int J Mol Sci. 2024 Jan 14;25(2):1032. doi: 10.3390/ijms25021032 (PMC10816441; doi:10.3390/ijms25021032)
Supplement: Supplementary file 1 [file ijms-25-01032-s001.zip › ijms-2776265-supplementary.pdf]

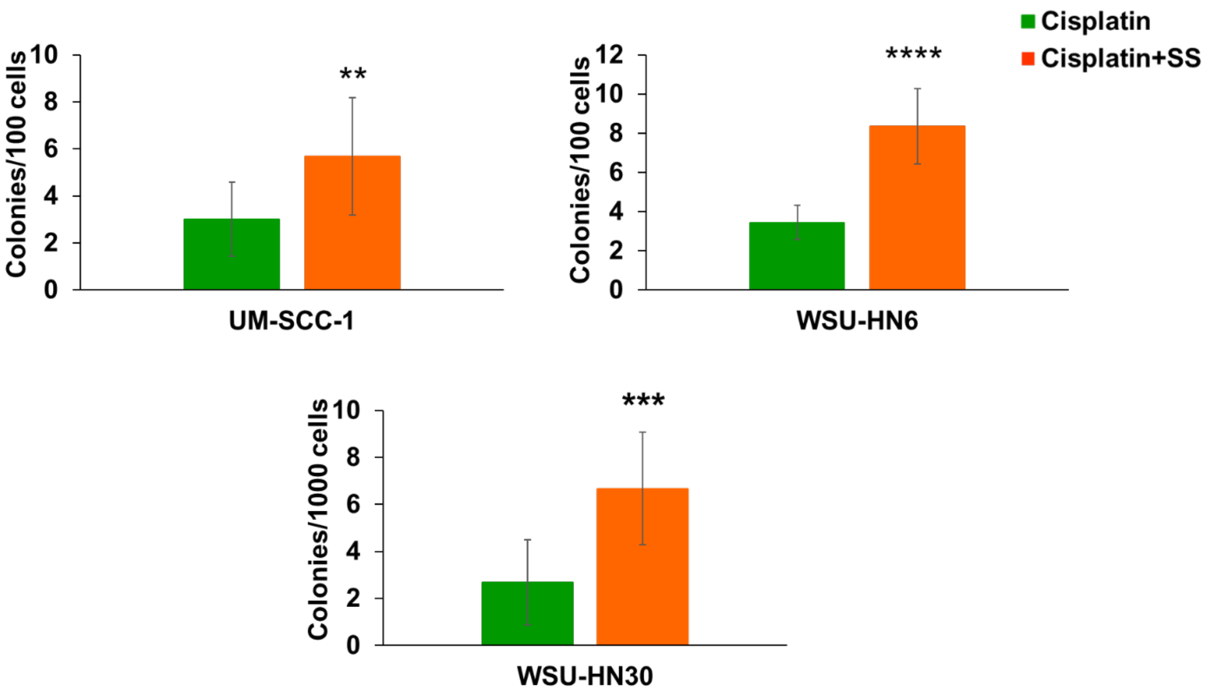

Figure S1. SS smoke extract exposure during cisplatin treatment for 24 hours increases cancer clonogenic survival. Indefinite reproductive viability of the cisplatin surviving cells was measured by counting the number of cell-forming colonies after 24 hours of treatment with cisplatin (2.5 μM) in the presence or absence of SS smoke extract. Data show that the presence of SS smoke extract significantly increases colony formation in all cell lines. Data shown as mean ± SD. $^{**}p<0.01$, $^{***}p<0.001$.

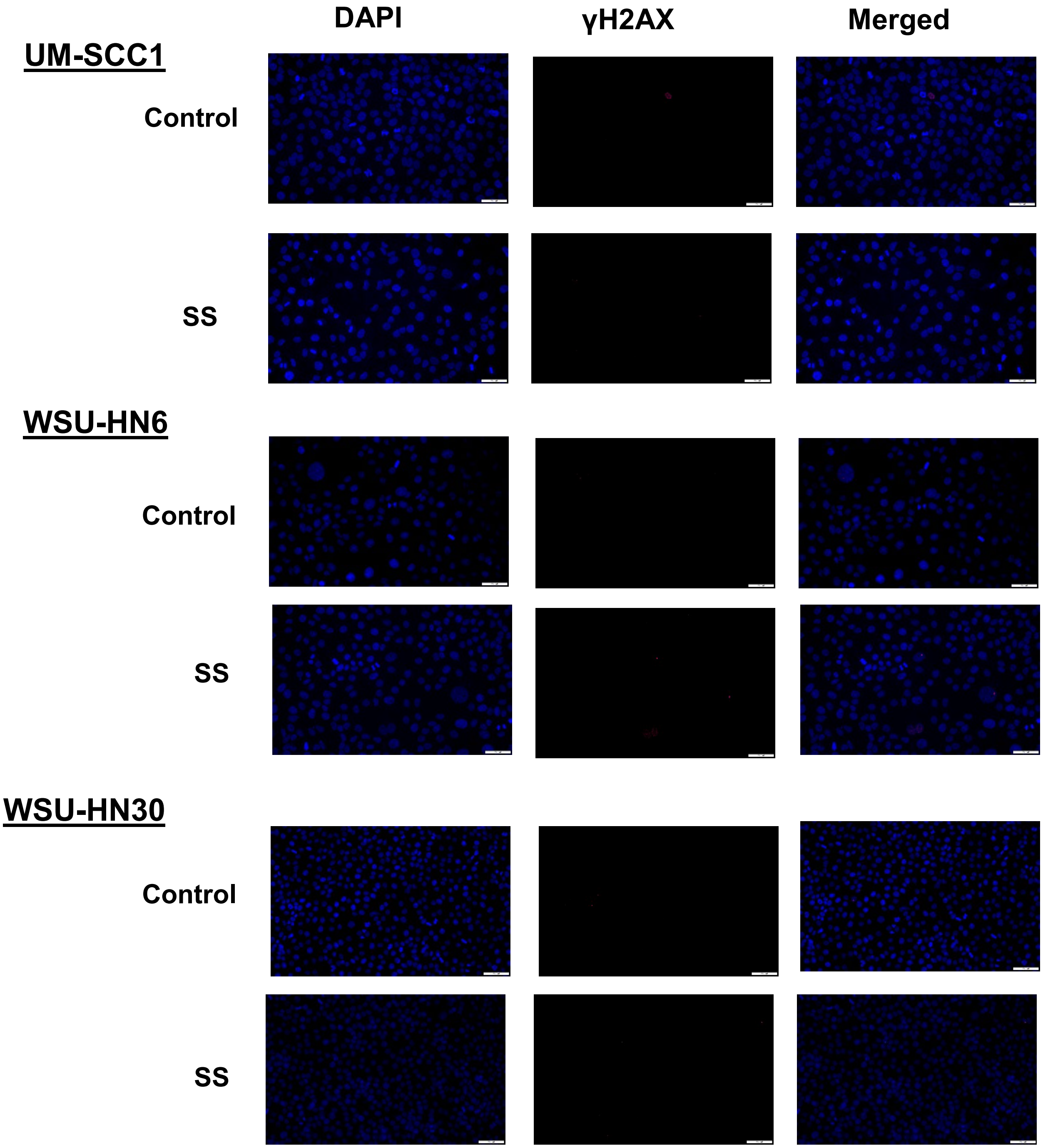

Figure S2. Images of γH2AX staining at 96 hours for all three cell lines. UM-SCC-1, WSU-HN6, and WSU-HN30 cells were exposed to SS smoke extract for 96 h. Double-strand breaks were quantified using γH2AX antibodies (bright, far-red, fluorescent dye) and DNA labelled with DAPI (blue). No difference was observed between SS-smoke-extract-only-exposed cells and unexposed control cells. The scale bar is 50μm.

ABGCG2 protein expression in HNSCC cells

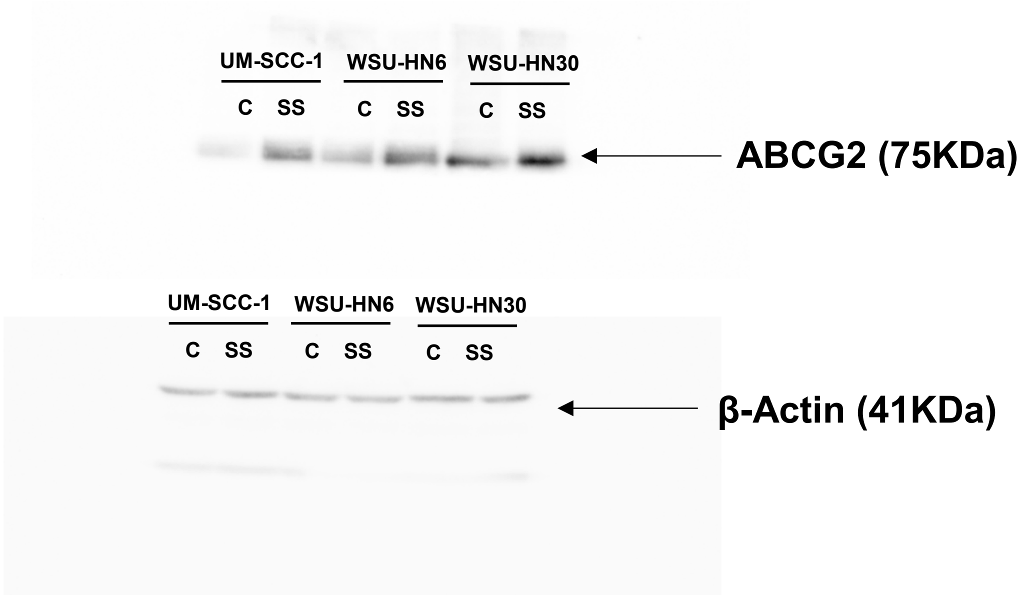

ATP7A protein expression in HNSCC cells

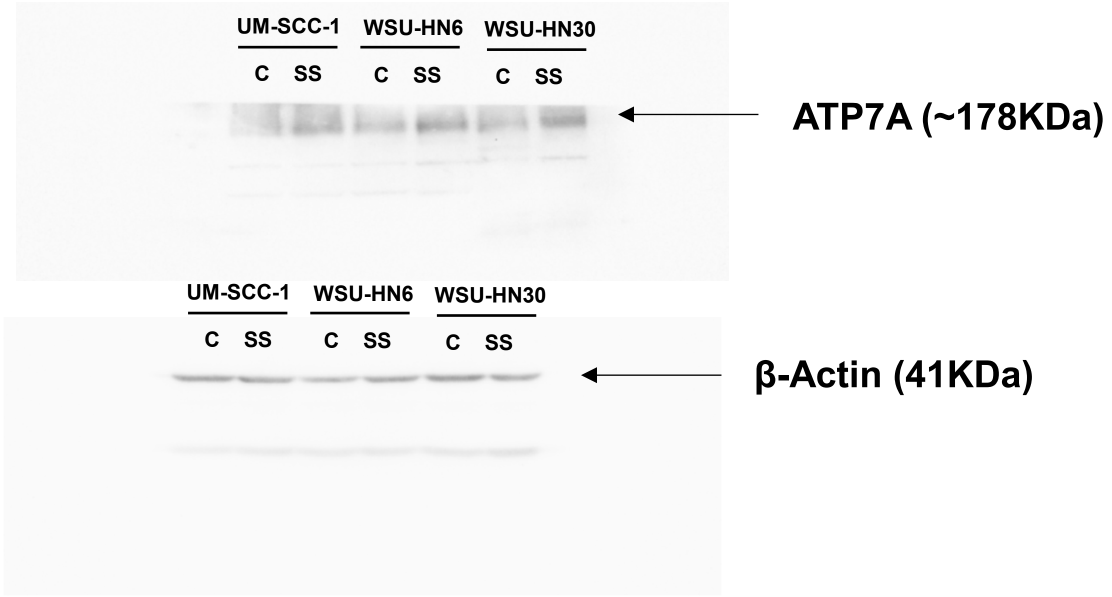

CTR1 protein expression in HNSCC cells

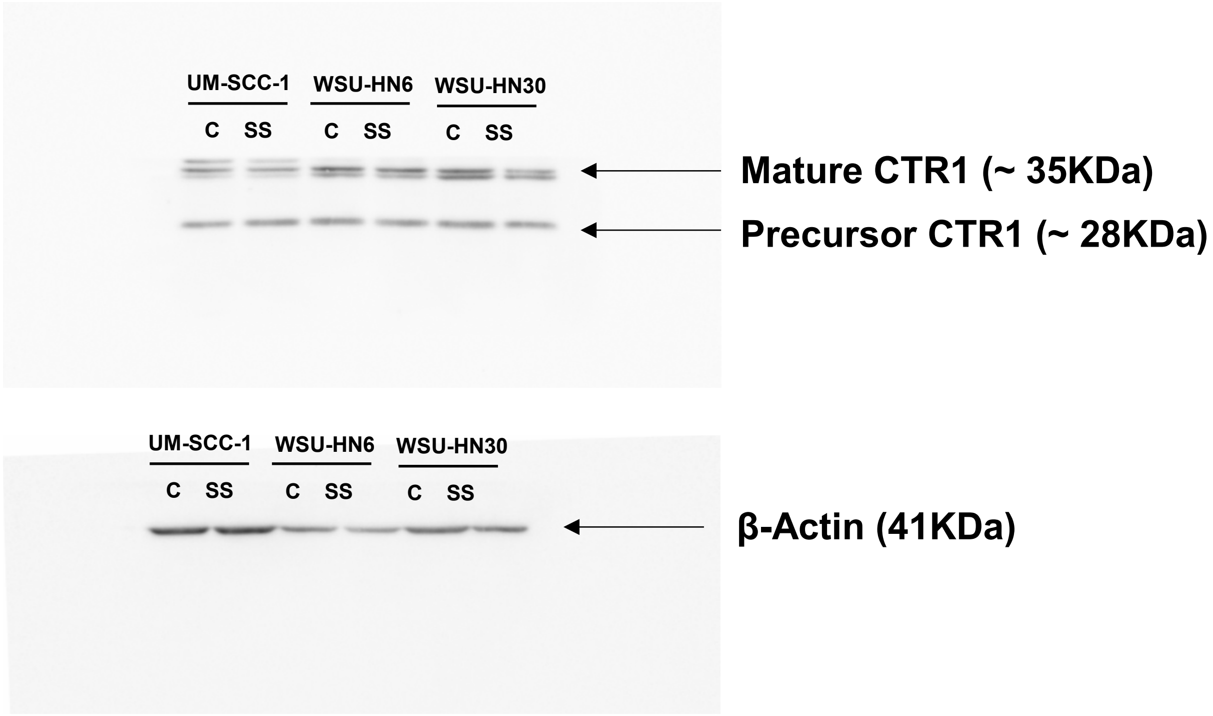

Original Western blots images for Figure 9. **Abbreviations:** SS- Side stream smoke extract; C- Control cells not-exposed to SS smoke extract.

	Manufacturer	Catalog #
**Primary antibody**		
anti-ABCG2	Cell Signaling Technology, Massachusetts, USA	42078
anti-ATP7A	Santa Cruz Biotechnologies, Texas, USA	sc-376467
anti-CTR1	Abcam, Massachusetts, USA	ab129067
β-actin	Cell Signaling Technology, Massachusetts, USA	8457
anti-Ki67	Cell Signaling Technology, Massachusetts, USA	9449S
anti-γH2AX	Sigma-Aldrich, Massachusetts, USA	05-636
**Secondary antibody**		
rabbit anti-mouse IgG	Abcam, Massachusetts, USA	ab6728
goat anti-Rabbit IgG	Cell Signaling Technology, Massachusetts, USA	7074
Goat anti-mouse IgG (Alexa Fluor@647)	Abcam, Massachusetts, USA	ab150119

Table S1. Antibodies used for Western blot and Immunofluorescence analysis.

Gene	Forward Primer	Reverse Primer
β-actin	5'-GTCATCACCATTGGCAATGAG-3'	5'-ATGTCCACGTCACACTTCATG-3'
ATP7A	5'-GCAGAGCCTCTATAAACTCAC-3’	5’-GTGTCATCATCTTCCCTGAAG-3'
CTR1	5'-CTCATCTTCATGACCTACAACG-3’	5’-GATGTCAATGGCAATGCTCTG-3'
ABCG2	5'-TGTGGCATTAAACAGAGAAGAAGAC-3'	5'-TCACCCCCGGAAAGTTGATG-3'
ERCC1	5’ -GGCGACGTAATTCCCGACT-3’	5’ -TAGCGGAGGCTGAGGAACA-3’
XPA	5'-GCAGCCCCAAAGATAATTGA-3'	5'-TGGCAAATCAAAGTGGTTCA-3'
MMS19	5'-GTCAGCAGGACCCTGAGAGTTC-3'	5'-CTCAGAACTGAGGGCTCCTTC-3'

Table S2. Primers used for mRNA expression analysis.
